# Closing the concern-action gap through relational climate conversations: insights from US climate activists

**DOI:** 10.1007/s44168-022-00027-0

**Published:** 2022-12-05

**Authors:** Julia Coombs Fine

**Affiliations:** Environmental Studies, College of St. Benedict & St. John’s University, St. Joseph, MN USA

**Keywords:** Climate change, Conversation, Relational, Climate justice, Activism, United States, Climate-change adaptation

## Abstract

Several studies have found that relational climate conversations can be an effective method of increasing conversational participants’ concern about the climate crisis and encouraging them to take collective action. However, little work has yet examined how such conversations are practiced by climate activists, a group with expertise in relational organizing. Drawing on surveys and semi-structured interviews with climate activists across the USA, this analysis finds that activists frequently have climate conversations with friends and family, most of whom are politically progressive and somewhat to very concerned about the climate crisis. These findings might seem to suggest that climate activists only have climate conversations with like-minded others, producing an echo chamber effect that could entrench the political polarization of the issue. However, climate activists report strategic reasons for choosing like-minded audiences, such as personal response efficacy. Additionally, they report that one of their primary conversational goals is to move people who are already concerned about the climate crisis to take collective action in accordance with values of climate justice. The results identify obstacles to collective climate action even among concerned audiences and suggest that relational climate conversations can be useful in overcoming these obstacles.

**Graphical Abstract:**

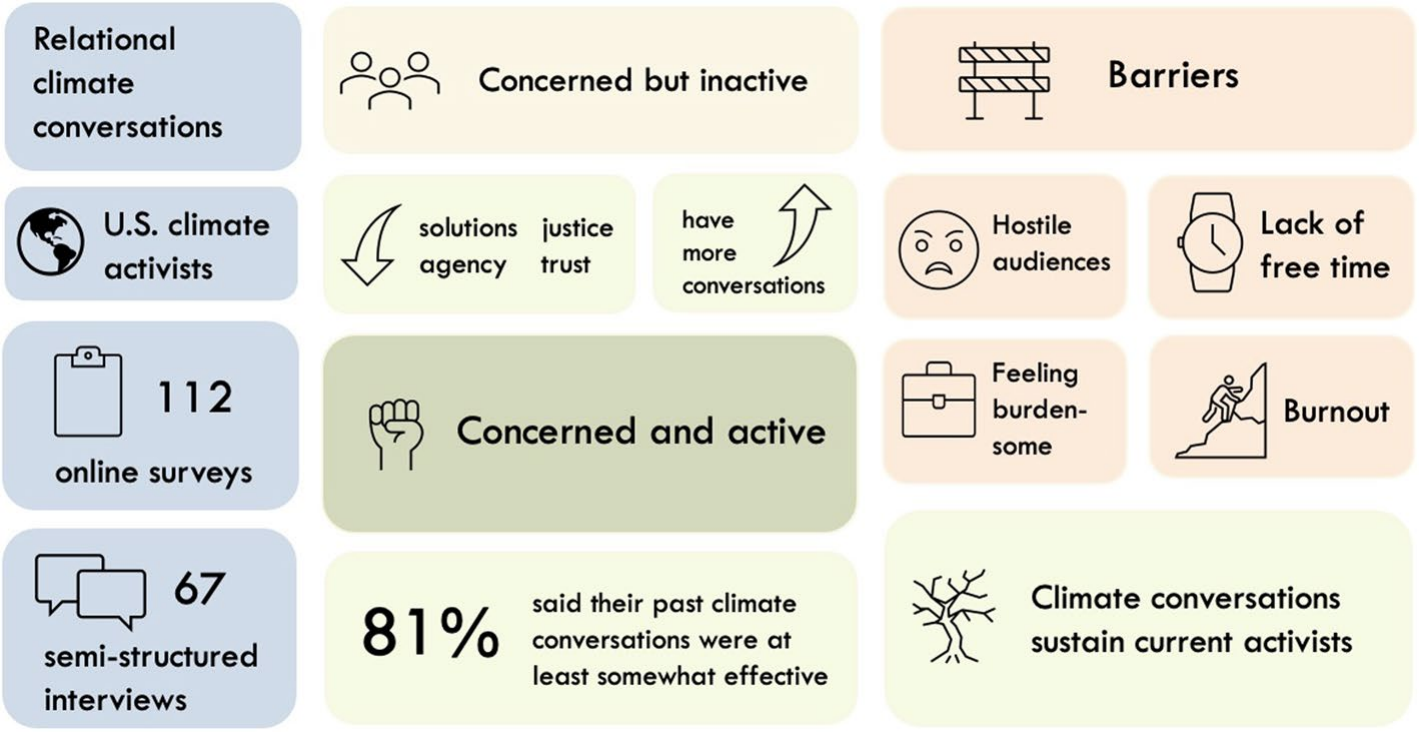

**Supplementary Information:**

The online version contains supplementary material available at 10.1007/s44168-022-00027-0.

## Introduction

As the effects of the climate crisis grow increasingly apparent, climate movements around the world have pushed for systemic change through tactics such as protests, strikes, boycotting, and direct action (Fisher & Nasrin, 2021:4). To support these efforts, many climate movements have embraced the strategy of relational organizing or intentionally fostering the interpersonal relationships that make up the fabric of social movements. “Climate conversations” have emerged as a particularly prominent form of relational organizing, with many organizations and climate communicators urging supporters to discuss climate change with their family and friends (Our Climate Our Future, n.d.; Hayhoe, 2018; Urry, 2016).

Relational climate conversations have been shown to shift participants’ climate attitudes and motivate behavior changes (Beery et al., 2019; Galway et al., 2021; Goldberg et al., 2019; Lawson et al., 2019). However, people in the USA, who are among the highest emitters of fossil fuels per capita (Ritchie et al., 2020), rarely discuss climate change with friends and family (Leiserowitz et al., 2019), resulting in a self-perpetuating climate of silence (Geiger & Swim, 2016). “Relational climate conversations” (Climate Advocacy Lab, n.d.) therefore deserve attention as an underused and potentially powerful tool for motivating climate action.

This analysis examines how climate activists, a group with expertise in relational organizing, use climate conversations as a strategy for outreach and movement building. The analysis draws on online surveys and interviews with climate activists across the USA to investigate their experiences with and recommendations for relational climate conversations. The results identify strengths and limitations of relational climate conversations and clarify areas in which they could be refined to improve their effectiveness as an organizing tool.

### Interpersonal climate conversations as relational organizing

Just and timely climate action is stymied by the dominance of wealthy, influential groups over everyday people in public policy (Caniglia et al., 2015:238). In part due to the association of climate change with science and politics, and the jargon surrounding these domains, many laypeople who are concerned about the climate crisis are estranged from the levers of power that would allow them to effect change (Kythreotis et al., 2019). This political disempowerment both arises from and contributes to a “socially constructed silence” around climate issues (Norgaard, 2011).

Community organizing is one means of empowering laypeople to have a greater voice in political decision-making (Rubin & Rubin, 2005). Under the umbrella of community organizing, relational organizing—creating and strengthening interpersonal relationships as a means of nurturing social movements—has gained prominence in climate movements worldwide (Divakaran & Nerbonne, 2017; Kowasch et al., 2021:18; Wahlström et al., 2019:12). As Han (2009) argues, relationships, as much or moreso than ideologies, motivate social movement participation. By cultivating relationships, climate activists can encourage others not only to mobilize for a short-term action but also to take on organizational responsibilities over the long term (Van Dyke & Dixon, 2013), a process known in many activist circles as the “ladder of engagement” (Hestres, 2015). This process may be driven by a sense of political solidarity grounded in feelings of community belonging (Cassegard et al., 2017). Relationships of trust and mutual respect can also have a protective effect on activists’ social and emotional well-being, supporting them to brave backlash and endure the psychological toll of the climate crisis (Bond et al., 2020:8-9).

One central tactic of relational organizing is interpersonal conversation (Grosse, 2019:8). This can take the form of one-on-one onboarding conversations between activists and new members (Lasoff, 2020) or conversations between current activists (Ospina & Foldy, 2010). Interpersonal climate conversations also occur between activists and their preexisting non-activist contacts, as encouraged by many organizations’ calls to “talk about climate change with friends and family” (see Greenpeace UK, 2021). Conversations that draw on preexisting relationships are relational in that they build on these relationships within the framework of climate organizing, thus strengthening climate movements (Climate Advocacy Lab, n.d.). In contrast, other interactive tactics, such as phonebanking and text-banking, form only fleeting connections between activists and audiences. Some tactics, such as deep canvassing (Fang, 2022), fall into a gray area, building stronger social connections during the interaction but not necessarily forging relationships that endure beyond it.

A small but promising body of research suggests that relational climate conversations can influence participants’ attitudes and behaviors in self-reinforcing ways. For instance, Beery et al. (2019):7-8) present evidence that a monthly climate change presentation and discussion series among environmental professionals encouraged participants to engage in climate action and have climate change conversations with others. Lawson et al. (2019) analyze climate conversations in the context of an intergenerational educational program conducted in North Carolina, finding that both children and parents who participated in the program became more concerned about climate change, with parents learning through their children rather than from teachers. This effect was particularly strong for politically conservative parents who were the least concerned about the climate crisis prior to the study. In a nationally representative study, Goldberg et al. (2019) found a reciprocal relationship between climate conversations and perceptions of scientific agreement: the more participants discussed climate change with friends and family, the more they became aware of the scientific consensus on climate change and vice versa. Furthermore, a postal survey of residents in Canada’s Provincial North found that higher levels of talking with family and friends about climate change were associated with greater engagement in climate action (Galway et al., 2021).

Despite their potential effectiveness, relational climate conversations are rare. According to Leiserowitz et al. (2019), only 6% of US residents report that they often discuss climate change with family and friends, and only 8% hear people they know talking about climate change once a week or more. The silence surrounding climate change engenders a self-perpetuating pattern of pluralistic ignorance: because people do not talk about climate change, they believe others are not concerned, which in turn makes them less likely to discuss it (Geiger & Swim, 2016). Relational climate conversations could disrupt this pattern of silence, engage non-activists in climate politics, and ultimately result in fairer and more rapid climate action.

### Target audiences for relational climate conversations

In theory, relational climate conversations could be used to reach a range of potential audiences with varied political orientations and climate attitudes. Previous research has focused extensively on the connection between conservative partisanship and climate dismissiveness, seeking ways to promote pro-climate attitudes among conservative audiences without producing boomerang effects (Bolsen & Shapiro, 2017; Hart & Nisbet, 2012; Zhou, 2016). Another preeminent conceptualization of potential audiences is the “Six Americas” model of audience segmentation (Leiserowitz et al., 2021), which divides Americans into six categories based on their attitudes towards the climate crisis: the alarmed, the concerned, the cautious, the disengaged, the doubtful, and the dismissive. Recent research has further segmented the alarmed category into the active alarmed (the 8% of the US population who engage in intensive climate action), the willing alarmed (the 11% who rarely engage in climate action but are willing to do so), and the inactive alarmed (the 5% who are unsure if they would participate in climate action) (Goldberg et al., 2021). The willing alarmed and the inactive alarmed add up to 16% of the population—the same percent who claim climate change is not occurring (Leiserowitz et al., 2019). Additionally, 12% of Americans reportedly believe that it is too late to take action against the climate crisis (ibid.), a viewpoint commonly known as “doomism” (Silva, 2022). The prevalence of inaction among the willing alarmed, inactive alarmed, and doomers indicates a concern-action gap (Never et al., 2020) that may be addressed through relational organizing, among other tactics.

Other audience selection considerations are specific to relational interactions. These considerations include the nature of the relationship between the participants, their degree of closeness, and activists’ efficacy beliefs about their ability to influence the audience’s climate attitudes and actions. Efficacy beliefs can be separated into self-efficacy (activists’ belief in their ability to take action) and personal response efficacy, also known as outcome expectancy (activists’ belief that their actions will produce the desired effects; see Bandura 1977, Kellstedt et al. 2008:118, and Doherty & Webler, 2016). We examine each of these relational factors alongside the more widely theorized considerations discussed above (political orientation and level of concern).

### Research questions

This study addresses the following research questions about climate activists’ prior experiences with climate conversations:

i. *Frequency*: How often do climate activists have climate conversations?
ii. *Contexts*: In which interactional contexts do they have climate conversations?
iii. *Barriers*: What factors prevent them from having climate conversations?
iv. *Audiences*: With whom do they discuss climate change?
v. *Confidence*: In which situations do they feel most and least confident talking about climate change?
vi. *Outcomes*: How have activists’ past climate conversations impacted the audiences with whom they spoke, as well as the activists themselves?

Additionally, the study examines the following questions about climate activists’ priorities for climate conversations:

i. *Target audiences*: Which target audiences do climate activists prioritize for climate conversations?
ii. *Goals*: What are their main goals for climate conversations?

## Methods

Participants were recruited through purposive sampling of climate-focused organizations across the USA, reaching out to at least one organization per state. The primary means of recruitment were emailing organizations; supplementary recruitment methods included snowball sampling and posting messages to email listservs, forums, and social media groups (Facebook, Twitter, Instagram, and Reddit). “Climate organizations” were defined as organizations that mentioned some form of climate action, such as political advocacy or education, in their mission statement or listed activities; it should be noted that this approach favors organizations that use explicit framings of climate action, such as many progressive groups, in contrast to conservative climate action groups that often couch their activities in other terms. Organizations with multiple participants included the Unitarian Universalist Ministry for Earth (9 participants), the Climate Reality Project (8 participants), 350.org (8 participants), the Sierra Club (6 participants), Citizens’ Climate Lobby (6 participants), the Sunrise Movement (6 participants), Ohio Youth for Climate Justice (5 participants), Power Shift Network (4 participants), the Pachamama Alliance (3 participants), Elders Climate Action (3 participants), The Climate Mobilization (2 participants), the Future Coalition (2 participants), Youth Climate Save (2 participants), and Maine Climate Action Now (2 participants). An additional 33 organizations participated, totalling 101 participating organizations. Most of these groups were 501c(3) or 501c(4) nonprofits or church groups, and their stated tactics largely centered on political advocacy (68 organizations) and education (49 organizations). More details about participating organizations can be found in Additional file 1: Appendix A.

The study was open to climate activists who lived in the USA and were at least 13 years old. The sample mostly consisted of volunteer climate activists and staff members of nonprofit climate organizations. Selected climate communication experts, such as narrative strategists, were also included. The sample was predominantly female and politically progressive, with a mean annual income range of US $25,000–US $49,000. The distribution of participants’ ethnoracial identities was similar to that of the US population overall. Most participants (88%) had been involved in climate organizing for at least 1 year, and most had also organized around related social justice issues, such as racial justice, Indigenous rights, and gender equality. For complete demographic information, please refer to Additional file 1: Appendix B.

In total, 112 online surveys and 67-h-long semi-structured conversational interviews were conducted in the 2021–2022. Participants had the option of participating in both the survey and the interview; 49 out of 67 interviewees also took the survey. The interview focused on similar questions to the survey but allowed participants to answer in more depth. To avoid double counting, results were not aggregated across the interviews and surveys. Due to concerns about the ethical implications of assigning pseudonyms (see Grinyer, 2009), participants were given the option of using their real names or pseudonyms of their choice. All real names presented in this study are shared with informed consent.

The surveys and interviews focused mainly on the following questions: (1) how frequently do US climate activists have climate conversations? (2) In what interactional contexts do they have them? (3) What are their target audiences? (4) What are their conversational goals? and (5) What outcomes have they observed from their past climate conversations? (see Additional file 1: Appendix C and Appendix D for the survey and interview instruments.) The interviewer was a white woman with 3 years of experience organizing with climate movements including the Sunrise Movement, Citizens’ Climate Lobby, and Extinction Rebellion. Demographic data was collected for interviewees via linked identifiers, for participants who also took the survey, and separate online forms, for those who did not. Chi-squared tests were used to test for independence between demographic variables and multiple-choice answers about climate activists’ experiences with and recommendations for climate conversations. Interviews and open response survey answers were analyzed in ATLAS.ti. The code list was generated through a combination of deductive and inductive methods, drawing on grounded theory (Strauss, 1987). First, we assigned a priori, category-level codes (e.g., “audiences”) to capture the themes raised by the interview questions and open-response survey questions. We then applied inductive codes within these categories (e.g., “moveable middle” as a subset of “audiences”) to capture emergent themes raised by participants. Finally, we refined a selection of the inductive codes most closely related to the research questions, combining overlapping categories. The complete list of codes and code descriptions is included in Additional file 1: Appendix E.

## Results

In section “Activists’ experiences with climate conversations: frequency, contexts, audiences, and barriers,” we discuss climate activists’ reported experiences with past climate conversations, including the frequency with which they had climate conversations, which interactional contexts they had them in, which audiences they engaged, and which barriers prevented them from discussing climate change more often. Section “Target audiences” focuses on activists’ recommended target audiences, and section “Conversational goals” focuses on their conversational goals. Finally, section “Outcomes of relational climate conversations” describes the reported outcomes of the conversations.

### Activists’ experiences with climate conversations: frequency, contexts, audiences, and barriers

Most participants reported having climate conversations in person, via email, or via social media (Table 1). Less commonly reported modes of communication included video chat, phone, and text chat. In contrast to the rarity of climate conversations among the general population (Leiserowitz et al., 2019), most respondents reported having climate conversations on a daily basis or several times per week, while we expected that climate activists would have climate conversations more often than the general public; this frequency is remarkably high. Participants most often reported having climate conversations at home, at work, and at climate-related demonstrations such as marches, vigils, and rallies. Less common contexts included religious organizations and schools. Most participants reported having conversations in small groups of 3–5 people or having one-on-one conversations. Medium group conversations, with 6–9 people, were also relatively common, as were large group conversations with 10 or more people.

**Table 1:** Modality, frequency, and context of climate conversations (survey responses, *N* = 111)

**Modality**	In person	Email	Social media	Video chat	Phone	Text chat
	90%	71%	61%	56%	40%	33%
**Frequency**	Daily	Several times per week	Once per week	Several times per month	Once every few months	
	41%	40%	10%	17%	2%	
**Context**	Home	Work	Demonstration	Religious organization	School	
	83%	65%	65%	40%	39%	
**Number of participants**	2 (one on one)	3–5 (small group)	6–9 (medium group)	≥ 10 (large group)		
	64%	72%	53%	55%		

Participants reported most often having climate conversations with fellow activists, friends, family members, political representatives, co-workers, and acquaintances (Table 2). Less frequently, they spoke with strangers and neighbors. Most participants reported having climate conversations with people who were somewhat or very concerned about climate change. However, 23% of participants reported having climate conversations with people who were slightly concerned, 11% reported having conversations with people who were not concerned, and 9% reported that they were unsure how concerned the people they talked to were. Most of their conversational audiences were somewhat progressive or very progressive. Thirty-two percent were neither progressive nor conservative, 25% were somewhat conservative, and 16% were very conservative.

**Table 2:** Audiences for climate conversations (survey responses, *N* = 111)

**Relationship**	Other activists	Friends	Family	Political representatives	Co-workers	Acquaintances	Strangers	Neighbors
	94%	80%	72%	65%	61%	58%	40%	27%
**Political affiliation**	Very conservative	Somewhat conservative	Neither progressive nor conservative	Somewhat progressive	Very progressive			
	16%	25%	32%	74%	66%			
**Concern level**	Not concerned	Slightly concerned	Somewhat concerned	Very concerned				
	11%	23%	67%	73%				

The most commonly reported barrier to having climate conversations was the possibility of conflict with climate skeptics and other hostile audiences. However, activists also mentioned many barriers to having climate conversations even with like-minded audiences. Commonly mentioned barriers within this subset included the COVID-19 pandemic, lack of free time, not wanting to be burdensome or intrusive, and feelings of burnout, such as futility and exhaustion (Table 3).

**Table 3:** Barriers to climate conversations

Barrier	Percent of responses	Example
Possibility of conflict	36%	*Fear of how it will be received has prevented me in the past, specifically with political ideology in mind.* -Respondent 96
COVID-19 pandemic	9%	*Of course*, *the COVID pandemic has been a big barrier in having face-to-face conversations with others about climate change.* -Respondent 45
Lack of free time	8%	*TIME. It takes TIME to listen and ask careful questions and really find out WHAT SPECIFICALLY they don’ know, which is SHAPING their opinion and attitude about the actions humans should or shouldn’t take.* -Respondent 37
Not wanting to be burdensome or intrusive	8%	*Not wanting to burden people who have personal issues that override their being able to process and act on any information that I might be able to offer.* -Respondent 2
Burnout	7%	*At times a feeling of futility or exhaustion.* -Respondent 50

### Target audiences

A majority of participants considered it most important to engage with people who were not sure what to think about climate change (77%) and who were not engaging with the topic (70%). Many participants also said it was important to talk to people who were somewhat concerned (57%) or very concerned (32%) about climate change. Fewer participants prioritized talking to people with views on the opposite end of the spectrum: 33% of participants considered it important to talk with people who were doubtful that climate change was happening, and only 19% of participants said that it was important to have climate conversations with people who were dismissive of climate change. The design of this question was influenced by the Yale Program on Climate Change Communication’s “Six Americas” study (Leiserowitz et al., 2021).

Participants’ prioritization of audiences who were somewhat concerned about climate change, not sure what to think, or not engaging with the topic aligns with the widely used concept of the “moveable middle,” i.e., those who neither strongly support nor strongly oppose a given issue; this concept corresponds to George Lakey’s “Spectrum of Allies” tool (Beautiful Trouble, n.d.). However, most participants (83%) also considered it important to target at least one group at the end of this spectrum, including people who were very concerned, doubtful, or dismissive about climate issues. Those who said it was important to reach out to doubtful and dismissive audiences often cited the US political system as a rationale for doing so. For example, interviewee Geoffrey DeSena, an activist with the Kootenai Environmental Alliance and Citizens’ Climate Lobby, noted that “We can’t get through a good comprehensive climate policy in Congress because there’s a vast middle of the country who are electing people who aren’t going to vote for anything like that. And so if we can’t talk to the vast middle of the country, then that is going to remain that way.” DeSena highlighted the need for respectful and open-minded dialogues with this demographic.

Several participants who considered it important to engage concerned but inactive audiences (the willing alarmed and the inactive alarmed in the YPCCC model) were interested in how to mobilize these audiences. Nine respondents mentioned the prevalence of concerned but inactive people in their networks, and in response to a solicitation of feedback on further research questions, two discussed a need for more research on how to influence this group to take action (1, 2)^1^.

(1) *How to get people who say they are concerned to take action. If that population began to take some action there would be significant progress*. -Respondent 3

(2) *I think figuring out how to get people who are somewhat or very interested in climate change to actually get involved. I see a lot of people who have interests and time but don’t actually do anything and it’s frustrating trying to think of ways to get them to get involved*. -Respondent 99

Excerpts 1 and 2 underscore that level of concern and interest (“people who say they are concerned,” “people who are somewhat or very interested in climate change”), level of current engagement in climate action (“but don’t actually do anything”), and capacity to take action (“have interests and time”) are best understood as three independent factors, each of which is relevant to audience selection.

### Low-hanging fruit: self-efficacy and personal response efficacy as factors in audience selection

Climate activists reported that their level of confidence in talking with various audiences also influenced their choice of target audience. Most commonly, they reported that they felt most confident talking to like-minded people such as educators, activists, and politically progressive people (44 responses; excerpt 3); people belonging to specific demographic groups, such as youth, women, working-class people, and Black and Indigenous people of color (14 responses; excerpt 4); open-minded and interested audiences (16 responses; excerpt 5); and close friends and family (10 responses; excerpt 6).

(3) *At work, or with family and friends – places where I already know there are like minds and the conversation is more about refining how to do the work*. -Respondent 8

(4) *I feel the most confident talking to women, youth, and BIPOC folks. I feel that they are receptive thinking about the social problems connected to climate change*. -Respondent 65

(5) *When I’m surrounded by open, receptive people who are willing to learn and have their perspective changed -- for instance, people who don't know that much about climate change and are open to learning more* -Respondent 79

(6) *With friends and loved ones. -*Respondent 10

In contrast, activists said they were least confident having climate conversations with people resistant to discussing climate issues (such as some conservative audiences,^2^ climate skeptics, doomers, and apathetic audiences) (41 responses; excerpt 7), people they did not know well (13 responses; excerpt 8), older adults, and audiences who are privileged along lines of race, gender, and socioeconomic class (6 responses; excerpt 9).^3^

(7) *Being in community with close minded conservative humans. If they’ve already made up their mind about something, why am I wasting my energy/time? -*Respondent 1

(8) *In a room of people who I know disagree with me, but whom I don’t know very well and don’t want to make angry. For instance a dinner party full of my parent’s friends who I know are conservative leaning. -*Respondent 70

(9) *White, middle/upper class people who think it is all about individual behavior change and who do not understand their privilege and judgmental attitudes. -*Respondent 76

Some participants reported that they hesitated to have climate conversations with strongly opposed audiences because they were concerned about possible boomerang effects (10, 11).

(10) *It sometimes feels useless. If the person is too opposed to the concept, I’m not a good enough debater to plant a seed of doubt. I’m also afraid that I might push them even further into their belief by pushing back too blatantly. My strengths lie in organizing, not debating or persuading, so I feel like my time would be better spent on something else rather than the conversation*. -Respondent 73

(11) *If I can tell someone is 100% not open to hearing about it, I typically don’t bother engaging because it won’t change their mind, just reinforce to them that People Like Me are wrong and bullish*. -Respondent 8

Excerpts 10 and 11 illustrate how both self-efficacy and personal response efficacy can influence audience selection: some activists feel that they lack conversation skills (e.g., “I’m not a good enough debater”) that would be necessary to engage opposed audiences (a problem of self-efficacy), while others perceive that the audience would not be swayed by anything they said (a problem of personal response efficacy).

To maximize personal response efficacy, many activists aimed for what one respondent termed “low-hanging fruit” (12).

(12) *I’d say that I don’t waste my time, energy or breath talking with those who are determined to misunderstand or lack interest in what I have to say. My experience has been to focus on “low hanging fruit”; those who are uncertain about the severity of the issue, how it affects others unlike themselves, and what steps can be taken to counter the crisis and improve their own resiliency and that of those they are in community with*. -Respondent 1

In this excerpt, respondent 1 conceptualizes “low-hanging fruit” as people who are unsure about the severity of the climate crisis, its effects, and avenues for action. This category aligns with doubtful and disengaged audiences (Leiserowitz et al., 2021) who are open to changing their views or in the terms used by activists in this study, open-minded audiences. Like-minded audiences, or the concerned and the willing alarmed (ibid.), constitute another group that could be considered low-hanging fruit in that they are relatively easy to persuade. In addition, activists’ attention to audiences’ race, gender, age, and socioeconomic status suggests that these factors may also determine openness to climate conversations.

### Social justice considerations in audience selection

Two interview participants reported that they did not prioritize conservative and white audiences, not because of efficacy considerations but because of social justice concerns. Public health expert and activist Monica Unseld commented that focusing on white conservative audiences could detract from engaging with frontline communities of color (13).

(13) *At this point, I’m not willing to meet the conservatives where they are. At this point. I’ve lost some white women friends over this, because I said, “You want to be the hero here and say that you converted some white people to your side. But all you really did was give them a platform to continue to spew lies and myths and hate, and you’ve centered whiteness there […] You’re like, ‘We have to reach out to this white community that may not understand, instead of Black and Brown people who maybe have their fourteenth cancer diagnosis […] .’ But you have decided that the priority is to convince white people to bring them along.” And I also think that’s a white savior characteristic*.

Here, Unseld challenges the dominant framing of “climate conversations” as outreach to audiences who are uninformed about or dismissive of climate issues and, in particular, conservative white people. Instead, she recommends reaching out to people of color who experience environmental injustice impacts in order to support them in developing community-led solutions.

Relatedly, Reem Skalli, the Columbus city lead for Ohio Youth for Climate Justice, noted that focusing on outreach to climate skeptics could create a hostile environment for frontline community members (15).

(15) *I think, if you have the capacity to reach out to [climate deniers], do it. But specifically in our organizing space, we prioritize the feelings and the safety of vulnerable communities. So we do not necessarily make it our number one priority to convert every climate denier or every right wing individual to our side, because that doesn’t seem like the most productive use of our time when we’re fighting this huge climate crisis already. […] That’s not to say that climate deniers aren’t valuable people. It’s just that they’re not on our list of people that we would prioritize bringing into our organization, because those aren’t necessarily the kind of people that are going to cultivate a safe community space for our other members*.

Skalli’s reflections reveal that organizational goals and culture inform the strategic use of climate conversations. While it may be advantageous for organizations focused on national policy change, such as the Citizens’ Climate Lobby, to reach out to climate skeptics who have disproportionate political influence in the electoral college, this strategy may be counterproductive for an organization working closely with frontline communities.

The above findings illustrate several factors that influence climate activists’ choice of target audiences for relational climate conversations, including audiences’ level of concern about the climate crisis, audiences’ level of engagement in climate action, activists’ efficacy in persuading audiences, activists’ organizational culture and goals, and the social justice implications of engaging privileged audiences over frontline communities.

Finally, it bears noting that the relationality of climate conversations provides an opportunity to engage audiences who may not initially be eager to discuss climate-related topics but who may become more open to such discussions as the relationship develops. Five interviewees pointed out that climate conversations are not typically isolated interventions but occur in an ongoing series with trust increasing over time. These relationships of trust were seen to be especially important for discussing weighty topics such as climate injustice. For instance, Alycia Bacon, a community activist with Mothers Out Front, commented, “My approach is focused on building relationships so that as we begin to have conversations that are more heavy, there’s already a sense of trust. Even if what I’m saying might be difficult to receive, you know that I am coming from a place of love and respect, and you can feel that sense of comfort that I’m talking to you as a person I care about and a person who has good intentions.”

### Conversational goals

According to survey responses, activists’ main goals for climate conversations included establishing basic understanding of the climate crisis, emphasizing the severity of climate impacts, informing people that climate change is human caused, conveying human agency to stop climate change, educating people about climate justice, and engaging people in collective climate action such as “strikes, lobbying, demonstrations, etc.” (Table 4). A less central but still important goal was to process audiences’ emotional reactions to the climate crisis. Fewer participants deemed it important to persuade audiences to make lifestyle changes (formulated in the survey question as “dietary changes, reducing air travel, etc.”) or to discuss their own emotional responses to the climate crisis.

- When you talk to people about climate change or climate action, how important to you are each of the following goals?

**Table 4:** Goals for climate conversations (survey responses, *N* = 111)

For them to	Not at all important	Slightly important	Somewhat important	Fairly important	Very important
Understand that the climate is changing	1%	9%	7%	24%	59%
Understand that climate change is caused by human activity	2%	5%	11%	21%	62%
Understand that climate change is a serious problem	1%	2%	7%	12%	78%
Understand the unjust causes and effects of climate change (for instance, that people of color and people in the Global South are disproportionately impacted)	3%	2%	12%	24%	60%
Understand that humans are capable of stopping climate change	1%	1%	5%	12%	82%
Make lifestyle changes (dietary changes, reducing air travel, etc.)	8%	19%	22%	22%	30%
Participate in collective action (strikes, lobbying, demonstrations, etc.)	3%	9%	17%	26%	45%
Discuss their emotional experiences of climate change	3%	12%	16%	32%	37%
Discuss your own emotional experiences of climate change	8%	15%	24%	28%	26%

Seventy-five percent of participants said that it was “very important” to convince their conversational partners to engage in climate action, including collective action (45%) and lifestyle changes (30%). When asked what kinds of climate conversations would be most useful to study in future research, furthermore, 18 survey respondents expressed interest in conversations that lead to climate action (16, 17).

(16) *Conversations between non-activists that nucleate new climate action* -Respondent 64

(17) *Moving beyond conversation to action...once we establish knowledge, how do we communicate the actions that they can take...and make them “stick.”* -Respondent 103

Interview data further reveal a high prioritization of social justice issues in climate conversations. While two interviewees noted that they sometimes led with other framings of climate issues for strategic reasons, fourteen interviewees emphasized that climate justice messaging was essential to building strong and equitable climate movements. For instance, narrative strategist and movement activist Patrick Reinsborough noted that addressing climate action as a justice issue is a way to form coalitions with communities that are most impacted by injustice (18).

(18) *At this point, if you’re not talking about justice as part of the climate movement, you’re missing a chance to connect with whole larger constituencies. There are still a lot of people who are maybe concerned about climate, but they’re actually busy fighting on other issues. They’re fighting around policing and racial justice issues in their community. They’re fighting around immigrant rights. They’re fighting to organize a union and have better economic justice. And if climate is still thought of as just an issue, then we’re competing. We’re telling those people, “Stop doing what you’re doing. You need to work on this.” As opposed to, climate justice creates a framework for this transition and helps folks understand the connection between a lot of these issues*.

Reinsborough further warned that by failing to emphasize climate justice, climate communicators may allow harmful narratives of the climate crisis to take hold: “If justice is not baked into the way we help people understand this, then we’re just doing the work potentially of fascists and racists and all of the other, more sinister forces that are also trying to narrate the changes around climate change.” Another participant reported that, in her experience organizing with the Sunrise Movement (a youth organization working towards climate justice), she successfully used intersectional and justice-oriented framings of climate change to engage “passive allies” (people who are already somewhat concerned about climate change and in favor of climate action) to become more active in climate organizing, commenting, “Using this human rights framing made them feel more passionate about the issue.” She estimated that one-third of the activists who were recruited through justice-focused relational organizing become long-term members. Her reflections demonstrate that, in addition to their inherent value (as discussed by Krantz and Reinsborough above), climate justice framings can be an effective way of motivating long-term action.

Interview data show that the above conversational goals may be pursued in tandem. For instance, Johannah Blackman, the executive director of the Maine-based organization A Climate to Thrive, described a conversational structure that begins by establishing foundational knowledge of the climate crisis and climate injustice, acknowledges and affirms emotional reactions to this crisis, and culminates in a call to action (19).

(19) *We thought this through as an organization ourselves and have come to really feel that there are three essential components in successful climate education. […] One is delivering scientific facts, and that’s where age appropriateness is really important. The second is holding space for emotional responses and validating that those responses are not only normal, they’re actually valuable—that it makes all the sense in the world, and is a valuable response, that we feel scared or sad when we hear about climate change. That points to the fact that something needs to be done and that we are dependent on the health of this planet. […] And then the third is providing meaningful avenues for participation and solutions. That’s both individual participation, like with groups, and also showing what solutions are being implemented by others. I think it’s really important that young people not feel like this has been a burden that’s being passed to them and that they need to carry by themselves*.

These reflections illustrate that climate activists intend climate conversations not just as a means of informing people of the basic facts about the climate crisis (such as its reality, its severity, and its origins in human activity) or about climate solutions (such as their existence and viability) but as a way to discuss the root causes of the climate crisis, process emotions, and motivate collective action.

### Outcomes of relational climate conversations

The outcomes of climate conversations between current climate activists and audiences can be separated into two components: the effects on audiences and the effects on climate activists themselves. In light of the prevalence of burnout and hopelessness among climate activists (Nairn, 2019), the effects of climate conversations on current climate activists merit serious consideration; otherwise, climate movements risk becoming revolving doors in which current activists burnout at the same rate that new activists are recruited.

#### Effects of climate conversations on audiences’ attitudes and behaviors

Overall, 73% of participants reported that their past climate conversations were at least somewhat effective at achieving their conversational goals for shifting audiences’ attitudes and actions. While only 9% of respondents reported that these conversations were “very” effective at achieving their conversational goals, 30% said they were “fairly” effective, 43% said they were “somewhat” effective, and 11% said they were “slightly” effective. Two percent said they were not effective, and 5% said they were unsure if they were effective. No participants said that their past climate conversations were counterproductive. Additionally, 37% of survey respondents named climate conversations as a central reason they themselves became involved in climate activism, suggesting that climate conversations are among the main factors that influence people to join climate movements.

Respondents further reported that their past climate conversations were more effective at influencing their conversational partners’ mindsets than their actions (Table 5).

**Table 5:** Effects of climate conversations on audience mindsets and actions

In general, to what extent have your past conversations about climate change affected	Not affected	Slightly affected	Somewhat affected	Fairly affected	Greatly affected
The mindsets of the people you have spoken with?	2%	12%	36%	**42%**	8%
The actions of the people you have spoken with?	3%	23%	**39%**	31%	4%

However, climate conversations did reportedly influence audiences’ actions, for the most part: only 3% of activists said that their previous climate conversations had not affected the actions of the people with whom they spoke. The most commonly noted impacts of climate conversations include taking political actions such as contacting representatives and voting (17 responses; excerpt 21), making lifestyle changes (16 responses; excerpt 20), and attending climate actions such as meetings, protests, and strikes (12 responses; excerpt 22).

(20) I’ve convinced some people to contact representatives, donate money, or attend actions who otherwise might not have bothered. -Respondent 8

(21) I’ve convinced several people to adapt a more ecologically sound diet. -Respondent 33

(22) Occasionally a friend has joined a climate action or attended a climate group meeting. -Respondent 5

Less commonly reported outcomes include audiences changing energy sources (e.g., installing solar panels or heat pumps) (5 responses; excerpt 23), demonstrating increased concern about the climate crisis (5 responses), and—perhaps most promisingly—becoming long-term climate activists (6 responses; excerpt 24).

(23) I’ve been able to turnout folks to their first actions & make activists out of concerned friends. -Respondent 64

(24) One friend went to the initial non-violent direct action training for the Society for Fearless Grandmothers^4^ and is now an active member of the group. -Respondent 5

In addition, 5 participants noted that their past climate conversations had influenced their conversational partners to educate themselves about climate issues (25) and 3 reported that their partners had had climate conversations with others (26).

(25) *People have told me that they haven’t been paying attention even though they know they need to learn and do more. After talking, they say they will learn and do more*. -Respondent 11

(26) *Some are willing to speak with their friends*. -Respondent 48

Participants further observed that their climate conversations helped to normalize talking about climate change. One participant commented that their past conversations had “chang[ed] the norm around climate change being a doomsday topic only fanatics talk about.” These results suggest that climate conversations can generate a positive feedback loop similar to the one found by Goldberg et al. (2019), prompting participants to seek more information about climate change and have additional conversations.

Despite these positive effects, participants emphasized the difficulty of mobilizing audiences to engage in collective action. For instance, the same participant who mentioned that they had inspired a friend to join the Society of Fearless Grandmothers observed that some of their past conversations had only increased their conversational partner’s knowledge and had not enabled them to take actions such as making public comments or attending rallies (27).

(27) *Mostly people are already very worried about climate and the future, but they feel helpless and uncertain about what to do. Sometimes they ask me to explain basic facts about climate change--so they end up a little more knowledgeable and even more apprehensive about the future but still unable to act. I tell them the most important thing they can do is talk about climate change with the people they know. I think they probably do talk more. Still, it seems hard for someone who has never done anything an activist would do to break through the boundaries they have set for themselves and actually make a public comment at a hearing or show up at a rally*. -Respondent 5

This participant’s reflections suggest that climate action can be especially daunting to those with no prior experience with activism. Similarly, 6 other participants identified a need for smaller, more approachable actions. Other commonly mentioned barriers to climate action included being overwhelmed with other priorities and stressors (23 responses; excerpt 27), not having enough free time to take action (17 responses; excerpt 28), efficacy beliefs (15 responses; excerpt 29), and the financial cost of participating in actions (15 responses; excerpt 30).

(27) *If someone is in survival mode, as so many are, it is difficult to care about something that is not of immediate concern like climate*. -Respondent 2

(28) *It’s hard for folks to volunteer to take individual action when people have to work full time (+) jobs and care for personal responsibilities as well*. -Respondent 55

(29) *People are doubtful that any actions they take will make a difference*. -Respondent 9

(30) *Typically barriers are socioeconomic. People who do care and want to take action cannot afford to take time from work or travel to do so*. -Respondent 69

Some of the above barriers to climate action, such as financial cost and lack of free time, may be difficult to overcome through climate conversations. However, others, such as overwhelm and efficacy beliefs, could be addressed. More work is needed to test strategies for identifying and overcoming these barriers in climate conversations.

#### Effects of climate conversations on current climate activists

Participants reported that climate conversations positively impacted their own attitudes and behaviors. Most participants (57%) said that having climate conversations made it easier for them to continue their involvement in climate action. Only 2% said that having climate conversations made it harder to keep taking action. An additional 18% of participants said having climate conversations did not make it easier or harder for them to continue taking action, 18% said it depended on the situation, and 4% said they were unsure. Participants who said climate conversations made it easier for them to continue taking action reported that these conversations could remind them of the need for action (15 responses; excerpt 31), lead to successful and inspiring outcomes (13 responses; excerpt 32), create a sense of community with others (9 responses; excerpt 33), allow them to learn new ideas and perspectives (5 responses; excerpt 34), increase their confidence in talking about climate issues (5 responses; excerpt 35), and help them to process their emotions in regard to the climate crisis (4 responses; excerpt 36).

(31) *When I get a positive reception, it reinforces that I’m doing the right thing and I have support; when I get a negative reception, it reinforces that there is a lot to work against and struggle is needed*. -Respondent 8

(32) *I feel inspired by people who decide that they need to take actions/change lifestyle/become active*.-Respondent 103

(33) *I enjoy the reminder we are not alone*. -Respondent 52

(34) *Talking with other folks often generates new ideas and perspectives that can regenerate the energy to organize around climate justice*. -Respondent 82

(35) *It builds my confidence in my knowledge and passion about the topic*. -Respondent 78

(36) *It makes it easier cause we can discuss the ways we experience eco- anxiety and climate grief. We can share in the collective anxiety and grief and from there move to a place of action and also healing*. -Respondent 10

Climate activists who said that climate conversations made it harder to continue taking action mentioned increased feelings of despair or grief (5 responses; excerpt 37), exhaustion (3 responses; excerpt 38), discouragement at others’ lack of knowledge (3 responses; excerpt 39), and disappointment at the lack of successful conversational outcomes (3 responses; excerpt 40).

(37) *It can be really overwhelming and at times feel hopeless to keep taking action and working hard when the scale of the disaster is so great and so many people are not taking it seriously*. -Respondent 51

(38) *I think maybe it makes it harder because I get so exhausted just talking about the problem*. -Respondent 9

(39) *When I hear educated people or even people who I would expect to be aware tell me that they didn’t know how dire things are or similar, and don’t even know the basics of what is happening, I get very discouraged*. -Respondent 11

(40) *They can be depressing when they don’t lead to any action or when they lead only to being disillusioning*. -Respondent 28

These results suggest that, one the one hand, conversations with open-minded and like-minded audiences can energize current activists to continue taking action and having conversations, resulting in a self-reinforcing effect. On the other hand, conversations with people who are apathetic or in denial can discourage current activists and make it harder for them to continue their own action. If burnout is a problem for an organization, therefore, it may be strategic to prioritize climate conversations with the audiences most likely to provide confidence and inspiration, e.g., interested audiences with whom activists have close relationships.

### Demographic correlations

We tested the relationship between demographic variables and activists’ reported experiences with and goals for climate conversations. Demographic variables tested included organizational role, time organizing, gender, race, age, income, political orientation, predominant political orientation of the surrounding area, and the degree to which each respondent was affected by climate injustice (calculated by assigning one point for agreeing with each of the following statements: “My community is disproportionately impacted by climate change,” “My community is not well represented in many environmental movements,” “I have personally been impacted by climate change,” “If a climate disaster occurred where I live, I would probably be in more danger than others,” and “If a climate disaster occurred where I live, I would probably face financial difficulty”). The analysis revealed the following correlations:

- Staff members reported having climate conversations more frequently than non-staff, *χ*^2^ (4, *N* = 111) = 16.7, *p* = 0.002.
- Younger respondents reported having climate conversations via social media more than older respondents, *χ*^2^ (3, *N* = 111) = 12.934, *p* = 0.005.
- Older participants reported having climate conversations at religious organizations more so than younger participants, *χ*^2^ (3, *N* = 111) = 29.4, *p* < 0.001.
- Activists with more organizing experience reported having climate conversations with strangers more often than newer activists, *χ*^2^ (3, *N* = 111) = 9.2, *p* = 0.027.
- Activists with more organizing experience reported having climate conversations with very politically conservative audiences more often than newer activists, *χ*^2^ (3, *N* = 111) = 11.8, *p* = 0.008.
- People living in very conservative areas thought it was important to have climate conversations with audiences who were not concerned about climate change, more so than people living in less conservative areas, *χ*^2^ (4, *N* = 111) = 18.6, *p* < 0.001.

Otherwise, we observed no significant correlations between demographic variables and activists’ experiences with and goals for climate conversations.

### Limitations of relational climate conversations

Based on the above results, relational climate conversations can be effective in increasing concern among open-minded and like-minded audiences but are less effective in supporting these audiences to take action. This suggests that more work is needed on (a) how to maximize the effectiveness of relational climate conversations (e.g., by placing more of a focus on opportunities for climate action) and (b) how to use them in combination with other action-focused communication strategies.

Another limitation of climate conversations relates to the problem of reach. While climate conversations are well-suited to consolidating support among audiences who are already somewhat concerned about climate change, who are close with climate activists, and who are open to discussing the topic, they may be less successful at reaching strangers, audiences who are not concerned, and audiences who are not willing to discuss it. Climate conversations with dismissive audiences, furthermore, can lead to boomerang effects and may demoralize or even endanger climate activists. Therefore, relational climate conversations may be best used in contexts that do not require engaging groups that are socially or ideologically estranged from climate activists or alongside tactics better suited to engaging these groups.

### Study limitations

It should also be noted that this study presents a limited window into US climate movements. More work is needed to investigate how climate activists’ goals for and experiences with relational climate conversations vary according to sociopolitical context, both within the USA and elsewhere (and particularly in the Global South). Further work could also probe into the experiences of climate activists who frame their work in other terms, such as public health advocacy or mutual aid networks. Additionally, this study did not touch on the influence of organizational trainings, goals, and supports on climate activists’ viewpoints on climate conversations; a consideration of these factors could shed further light on activists’ priorities for climate conversations. Finally, these results only give insight into climate activists’ reports of the outcomes of climate conversations. Future research could examine how non-activist conversational participants perceive these outcomes or directly measure conversational outcomes by tracking participants’ engagement in online climate actions.

## Conclusion

This analysis has shown that US climate activists identify “low-hanging fruit”—people who are already somewhat concerned about the climate crisis, who are open to discussing it, and who have close relationships with activists—as an important target audience for climate conversations. Climate conversations can promote concern and understanding among this audience and can influence them to seek out more information, have subsequent conversations with others, and participate in collective actions such as protests. In addition, climate conversations help to sustain current activists’ involvement in climate movements.

However, activists also highlight difficulties in engaging open-minded and even like-minded audiences. Audiences are overwhelmed by other stressors, such as racial and economic injustice; this is particularly true of those most affected by the climate crisis. Furthermore, activists feel social pressure not to talk about climate issues, fearing that they will be perceived as burdensome, judgmental, or ignorant. Faced also with the uphill struggle of organizing and the enormity of the climate crisis itself, activists often wrestle with exhaustion and feelings of futility. Moreover, their conversational goals—conveying the seriousness of the climate crisis, convincing audiences of the existence of viable solutions, discussing climate justice, and inviting audiences to participate in collective action—are ambitious in a national context saturated with messages that the climate crisis is either nonexistent, faraway, or inevitable. In particular, activists report that promoting action is harder than changing audience mindsets, though they report some success cases of inspiring sustained collective action.

These results add to the emerging body of literature indicating that relational climate conversations can be an effective way of influencing audiences’ attitudes and, albeit to a lesser extent, actions. The results further highlight the need to engage concerned but inactive audiences and the value of climate conversations as a means for doing so. As concern about the climate crisis rises across nations (Gaffney et al., 2021), relational climate conversations could be a key strategy for consolidating support for climate solutions. More work is needed to further refine the potential of relational climate conversations as an organizing tool, not only in the USA but also worldwide.

## Supplementary Information

**Additional file 1:** **Appendix:** **Appendix B.** Demographic information of interview and survey participants. **Appendix C.** Survey instrument. **Appendix D.** Interview questions. **Appendix E.** Code list.

## Data Availability

The interview data associated with this study is available through (data repository name and link).

## Footnotes

1 Quotes are numbered in parentheses throughout the analysis to enable readers to refer to specific quotes; survey numbers are provided to enable readers to differentiate between respondents.

2 Though climate skepticism is much more rampant among conservative than progressive people in the US context (Wong-Parodi & Feygina 2020:61), it should be noted that not all conservative people are climate skeptics.

3 Some participants, however, noted that they felt less confident having conversations with close family and friends because they had demonstrated hostility or lack of interest in the subject.

4 The Society for Fearless Grandmothers is a Santa Barbara-based organization that trains older women to participate in climate action, https://www.fearlessgrandmotherssb.org/.
