# Supplementary material for: Closing the concern-action gap through relational climate conversations: insights from US climate activists
Source: Clim Action. 2022 Dec 5;1(1):26. doi: 10.1007/s44168-022-00027-0 (PMC9734534; doi:10.1007/s44168-022-00027-0)
Supplement: Supplementary file 1 — Additional file 1: Appendix: Appendix B. Demographic information of interview and survey participants. Appendix C. Survey instrument. Appendix D. Interview questions. Appendix E. Code list. [file 44168_2022_27_MOESM1_ESM.docx]

Appendix A: Participating organizations

| **Organization** | **No. of respondents** | **Region** | **Action focus** | **Shared identity of participants** | **Organization type** |
| --- | --- | --- | --- | --- | --- |
| 350 | 8 | International | Political advocacy | n/a | 501c(3) nonprofit |
| Agri-Tech Producers, LLC | 1 | South | Technological solutions | n/a | LLC |
| AMP Creeks Council | 1 | South | Education, legal action | n/a | Not specified |
| Arizona Youth Climate Coalition | 1 | Southwest | Political advocacy, education | Youth | Not specified |
| Aytzim: Ecological Judaism | 1 | International | Political advocacy | Jewish | 501c(3) nonprofit |
| Bvlbancha Collective | 1 | South | Mutual aid | Indigenous | Not specified |
| Care About Climate | 1 | International | Education, political advocacy | Youth | Not specified |
| Catholic Climate Covenant | 1 | National | Education, political advocacy | Catholic | 501c(3) nonprofit |
| Center for an Ecology Based Economy | 1 | Northeast | Education, political advocacy | n/a | 501c(3) nonprofit |
| Citizens Climate Lobby | 6 | International | Political advocacy | n/a | 501c(4) nonprofit |
| Clean Wisconsin | 1 | Midwest | Legal action, political advocacy, education | n/a | 501c(3) nonprofit |
| Climate Action Campaign | 1 | West | Political advocacy | n/a | 501c(3) nonprofit |
| Climate Action CdA (Coeur d'Alene, ID) | 1 | Midwest | Education, political advocacy | n/a | Not specified |
| Climate Action Families | 1 | Pacific Northwest | Education, political advocacy | n/a | 501c(3) nonprofit |
| Climate Action team at our UU Church | 1 | South | Not specified | Unitarian Universalist | Church group |
| Climate Generation | 1 | Midwest | Education, political advocacy | n/a | 501c(3) nonprofit |
| Climate Science Roadshow | 1 | Not specified | Education | Not specified | Not specified |
| Climate Solutions Accelerator of Genesee-Finger Lakes Region | 1 | Northeast | Education, political advocacy | n/a | 501c(3) nonprofit |
| Coming Clean | 1 | National | Political advocacy | n/a | Coalition |
| Compost Santa Barbara | 1 | West | Green infrastructure services | n/a | Volunteer group |
| Conejo Climate Lobby | 1 | West | Education, political advocacy | n/a | Not specified |
| Daily Acts | 1 | West | Education, political advocacy | n/a | 501c(3) nonprofit |
| Democratic Socialists of America | 1 | National | Political advocacy | n/a | 501c(4) nonprofit |
| Earth Action, Inc. | 1 | International | Political advocacy | n/a | 501c(3) nonprofit |
| Earth Guardians | 1 | International | Education, legal action, political advocacy | Youth | 501c(3) nonprofit |
| EarthCorps | 1 | Pacific Northwest | Education, ecological restoration | n/a | 501c(3) nonprofit |
| Eco Vista | 1 | West | Education, green infrastructure services, mutual aid | n/a | Not specified |
| Elders Climate Action | 3 | National | Education, political advocacy | Elders | 501c(3) nonprofit |
| Environmental Affairs Board | 1 | Not specified | Not specified | Not specified | Student organization |
| Environmental Justice Ministry with my church (All Souls Community Church of West Michigan) | 1 | Midwest | Political advocacy, green infrastructure services | Unitarian Universalist | Church group |
| Faith Communities Environmental Network | 1 | Northeast | Education, political advocacy | n/a | 501c(3) nonprofit |
| Florence Climate Emergency Campaign | 1 | West | Political advocacy | n/a | Not specified |
| Florence Indivisible | 1 | National | Political advocacy | n/a | 501c(4) nonprofit |
| Food Not Bombs | 1 | International | Mutual aid, green infrastructure services | n/a | 501c(3) nonprofit |
| Fridays for Future | 1 | International | Political advocacy | Youth | Not specified |
| Friends Committee on National Legislation | 1 | National | Political advocacy | Quaker | Not specified |
| Future Coalition | 2 | National | Education, political advocacy | Youth | 501c(3)/501c(4) nonprofit |
| Generation Atomic | 1 | National | Education, political advocacy | n/a | 501c(3) nonprofit |
| Green Faith | 1 | International | Education, political advocacy, divestment | n/a | 501c(3) nonprofit |
| Green Team for my UU congregation | 1 | Not specified | Not specified | Unitarian Universalist | Church group |
| Gulf Coast Creation Care | 1 | South | Education | Interfaith | Not specified; Coalition |
| Hike the Divide | 1 | n/a | Education | n/a | Not specified |
| Hometown Action | 1 | South | Education, political advocacy | n/a | 501c(4) nonprofit |
| Kentucky Conservation Committee | 1 | South | Political advocacy | n/a | Not specified |
| Louisville Climate Action Network | 1 | South | Education, political advocacy | n/a | Not specified |
| Maine Climate Action Now | 2 | Northeast | Education, political advocacy | n/a | Not specified |
| Millennials 4 Environmental Justice | 1 | National | Education, political advocacy | Youth | Not specified |
| Mobile Baykeeper | 1 | South | Education, political advocacy | n/a | Not specified |
| Mothers Out Front | 1 | National | Education, political advocacy | Mothers | 501c(3) nonprofit |
| MoveOn | 1 | National | Education, political advocacy | n/a | 501c(4) nonprofit |
| Mount Vernon Unitarian Church Climate Action Team | 1 | South | Not specified | Unitarian Universalist | Church group |
| My church group | 1 | Not specified | Not specified | Faith (not otherwise specified) | Church group |
| National Audubon society | 1 | National | Education, political advocacy | n/a | 501c(3) nonprofit |
| New Haven Climate Movement | 1 | Northeast | Education, political advocacy | Youth | Not specified |
| New Haven Leon Sister City Project | 1 | International | Education, sustainable development | n/a | Not specified |
| New Jersey Organizing Project | 1 | Northeast | Political advocacy | n/a | 501c(4) nonprofit |
| New Jersey Resource Project | 1 | Northeast | Not specified | n/a | Not specified |
| Ohio Youth for Climate Justice | 5 | Midwest | Political advocacy | Youth | Not specified |
| Okla Hina Ikhish Holo | 1 | South | Continuing cultural traditions | Femme/non-binary, Indigenous | Not specified |
| Our Future Program | 1 | Not specified | Not specified | Not specified | Not specified |
| Oxfam America | 1 | International | Political advocacy | n/a | 501c(3) nonprofit |
| Pachamama Alliance | 3 | International | Legal action, education, political advocacy | n/a | 501c(3) nonprofit |
| Partners for Sustainable Schools | 1 | West | Education | n/a | 501c(3) nonprofit |
| Partners for the Environment | 1 | Midwest | Education, political advocacy | n/a | Not specified |
| Physicians for Social Responsibility | 1 | National | Political advocacy | Doctors | 501c(3) nonprofit |
| Pittsburgh Freethought Coalition | 1 | Northeast | Education | n/a | 501c(3) nonprofit |
| Poor People's Campaign | 1 | National | Political advocacy | n/a | 501c(3) nonprofit |
| Power Shift Network | 4 | National | Education, political advocacy | n/a | 501c(3) nonprofit; coalition |
| PowerSwitch Action | 1 | National | Research, political advocacy | n/a | 501c(3) nonprofit; coalition |
| Problems, Predicaments, and Technology | 1 | Not specified | Education | n/a | Not specified |
| RE-AMP | 1 | Midwest | Research, political advocacy | n/a | Coalition; not specified |
| Santa Barbara County Action Network | 1 | West | Political advocacy | n/a | 501c(3) nonprofit |
| Save Diablo Canyon | 1 | West | Political advocacy | n/a | Not specified |
| Sierra Club | 6 | National | Education, legal action, political advocacy | n/a | 501c(4) nonprofit |
| Society of Fearless Grandmothers Santa Barbara | 1 | West | Political advocacy | Elder women | Not specified |
| Stand.earth | 1 | International | Divestment, political advocacy, legal action | n/a | 501c(3) nonprofit |
| Student Leadership for Environmental Action Fund | 1 | West | Education, political advocacy | Youth | Student organization |
| Sunrise Movement | 6 | National | Education, political advocacy | Youth | 501c(3)/501c(4) nonprofit |
| Talk Climate | 1 | Not specified | Not specified | Not specified | Not specified |
| The Climate Mobilization | 2 | National | Political advocacy | n/a | Not specified |
| The Climate Reality Project | 8 | International | Political advocacy | n/a | 501c(3) nonprofit |
| The Red Black and Green New Deal | 1 | National | Political advocacy | Black, Indigenous | Not specified |
| Unitarian Universalist Community Church of Santa Monica | 1 | West | Not specified | Unitarian Universalist | Church group |
| Unitarian Universalist Congregation | 1 | Not specified | Not specified | Unitarian Universalist | Church group |
| Unitarian Universalist Congregation of Columbus, IN | 1 | Midwest | Not specified | Unitarian Universalist | Church group |
| Unitarian Universalist Ministry for Earth | 9 | National | Education, political advocacy | Unitarian Universalist | Not specified |
| Unitarian Universalist Service Committee (Climate Justice) | 1 | International | Political advocacy, humanitarian aid | Unitarian Universalist | 501c(3) nonprofit |
| Unitarian Universalists for Just Economic Community | 1 | National | Political advocacy | Unitarian Universalist | Not specified |
| Unitarian Universalists for Social Justice | 2 | National | Education, political advocacy | Unitarian Universalist | 501c(3) nonprofit |
| United Church of Christ | 1 | National | Education, political advocacy | Christian | 501c(3) nonprofit |
| Until Justice Data Partners | 1 | South | Research | n/a | 501c(3) nonprofit |
| Uplift | 1 | Southwest | Education, political advocacy | Youth | 501c(3) nonprofit |
| USNH Green Sanctuary | 1 | Northeast | Not specified | Unitarian Universalist | Church group |
| UU Church | 1 | Not specified | Not specified | Unitarian Universalist | Church group |
| UU Church of Arlington | 1 | South | Not specified | Unitarian Universalist | Church group |
| UU Falmouth | 1 | Northeast | Not specified | Unitarian Universalist | Church group |
| UUCC | 1 | South | Not specified | Unitarian Universalist | Church group |
| Washington Native Plant Society | 1 | Pacific Northwest | Conservation, education | n/a | 501c(3) nonprofit |
| Women's Earth Alliance | 1 | International | Education, political advocacy | Women | 501c(3) nonprofit |
| Youth Climate Save | 2 | International | Education, political advocacy | Youth | Not specified |
| Youthpower Climate Action team | 1 | Midwest | Political advocacy | Youth | Not specified |

Appendix B: Demographic information of interview and survey participants

| **Gender** | **Percent of survey participants** | **Percent of interviewees** |
| --- | --- | --- |
| Female | 56% | 52% |
| Male | 30% | 29% |
| Genderqueer/Non-binary/Agender | 14% | 19% |

| **Race** | **Percent of survey participants** | **Percent of interviewees** | **National average (2020)^[[1]](#footnote-1)^** |
| --- | --- | --- | --- |
| White | 67% | 57% | 62% |
| Black, African American, or North African | 13% | 16% | 14% |
| Asian | 7% | 8% | 7% |
| Multiracial | 6% | 10% | 10% |
| Latine | 5% | 6% | 18.7% |
| American Indian, Alaska Native, Indigenous, or Indigenous and Chicana | 2% | 3% | 2.9% |

| **Age range** | **Percent of survey participants** | **Percent of interviewees** |
| --- | --- | --- |
| 0-17 | 6% | 6% |
| 18-24 | 18% | 25% |
| 25-34 | 19% | 22% |
| 35-44 | 7% | 8% |
| 45-54 | 6% | 6% |
| 55-64 | 16% | 14% |
| 65-74 | 18% | 10% |
| 75-84 | 7% | 6% |
| 85-94 | 2% | 0% |
| 95+ | 0% | 2% |

| **State of residence** | **Percent of survey participants** | **Percent of interviewees** |
| --- | --- | --- |
| California | 16% | 14% |
| Colorado | 8% | 6% |
| Ohio | 6% | 6% |
| Washington | 6% | 8% |
| Alabama | 5% | 2% |
| Michigan | 5% | 5% |
| New York | 5% | 10% |
| Illinois | 4% | 2% |
| Arizona | 3% | 8% |
| Connecticut | 3% | 3% |
| Florida | 3% | 2% |
| Indiana | 3% | 3% |
| Maine | 3% | 2% |
| Maryland | 3% | 3% |
| New Jersey | 3% | 3% |
| Oregon | 3% | 3% |
| Virginia | 3% | 5% |
| Kentucky | 2% | 3% |
| Minnesota | 2% | 0% |
| Missouri | 2% | 0% |
| North Carolina | 2% | 0% |
| Pennsylvania | 2% | 0% |
| Texas | 2% | 0% |
| DC | 1% | 2% |
| Delaware | 1% | 0% |
| Georgia | 1% | 2% |
| Idaho | 1% | 0% |
| Iowa | 1% | 0% |
| Louisiana | 1% | 3% |
| Massachusetts | 1% | 0% |
| New Mexico | 1% | 2% |
| South Carolina | 1% | 0% |
| Utah | 1% | 2% |
| Wisconsin | 1% | 3% |

| **Political orientation** | **Percent of survey participants** | **Percent of interviewees** |
| --- | --- | --- |
| Very progressive | 73% | 75% |
| Somewhat progressive | 18% | 11% |
| Neither progressive nor conservative | 2% | 0% |
| Somewhat conservative | 2% | 2% |
| Very conservative | 1% | 2% |
| Other | - | 6% |
| Prefer not to answer | 5% | 5% |

| **Annual income range** | **Percent of survey participants** | **Percent of interviewees** |
| --- | --- | --- |
| $0 | 2% | 0% |
| $1-$9,999 | 10% | 6% |
| $10,000-$24,999 | 18% | 20% |
| $25,000-$49,999 | 24% | 30% |
| $50,000-$74,999 | 26% | 28% |
| $75,000-$99,999 | 7% | 10% |
| $100,000-$149,999 | 12% | 4% |
| $150,000 or more | 1% | 2% |

| **Climate change impactedness and marginalization** | **Percent of survey respondents** | **Percent of interviewees** |
| --- | --- | --- |
| My community is disproportionately impacted by climate change | 36% | 40% |
| My community is not well represented in many environmental movements | 46% | 46% |
| I have been personally impacted by climate change | 56% | 65% |
| If a climate disaster occurred where I live, I would probably be in more danger than others | 17% | 22% |
| If a climate disaster occurred where I live, I would probably face financial difficulty | 35% | 38% |

Additional demographic information (survey participants)

| **Time organizing** | **Percent of survey participants** |
| --- | --- |
| Less than 1 year | 12% |
| 1-2 years | 24% |
| 3-5 years | 23% |
| 5 or more years | 41% |

| **Related organizing experience** | **Percent of survey participants** |
| --- | --- |
| Racial justice | 77% |
| Indigenous rights | 72% |
| Gender equality | 45% |
| Labor justice | 43% |
| Immigration rights | 36% |
| LGBTQIA+ rights | 28% |
| Disability justice | 23% |
| Other | 6% |

| **Organizational role^[[2]](#footnote-2)^** | **Percent of survey participants** |
| --- | --- |
| Volunteer | 58% |
| Staff | 33% |
| Board member | 14% |
| Director, founder, or group leader | 13% |

Appendix C: Survey instrument

**Background information: Climate justice organizing**

In this first section, we’re interested in learning about your involvement with climate justice organizing. By “climate justice organizing,” we mean work that addresses issues such as racial justice, gender equality, and labor justice in connection with climate change and prioritizes the leadership of frontline communities.

Are you involved with any organizations working towards climate justice? If so, which organization(s)?

What role do you play in the organization(s)?

*Please select all that apply.*

|  | Staff |
| --- | --- |
|  | Volunteer |
|  | Board member |
|  | Other: |
|  |  |

How long have you been involved in climate justice organizing?

|  | Less than 1 year |
| --- | --- |
|  | 1-2 years |
|  | 3-5 years |
|  | 5 or more years |
|  |  |

What motivates you to be an organizer?

What are some of the main goals of your organization?

How does your organization plan to achieve these goals? (i.e., what is their theory of change?)

Which of the following social justice concerns have you worked on in connection to climate change?

*Please select all that apply.*

|  | Gender equality |
| --- | --- |
|  | Disability justice |
|  | Indigenous rights |
|  | Immigration rights |
|  | Labor justice |
|  | LGBTQIA+ rights |
|  | Racial justice |
|  | Other: |

Which of the following types of climate change communication most inspired you to get involved in climate justice organizing?

*Please select up to three.*

|  | Attending a climate event |
| --- | --- |
|  | Books |
|  | Classroom education |
|  | Conversations with family and friends |
|  | Documentary films |
|  | Emails or ads from organizations |
|  | News articles |
|  | Op-eds |
|  | Scientific articles |
|  | Speeches by climate activists |
|  | Other: |

**Climate conversations: Experiences**

Over the past year, about how frequently have you had conversations about climate change or climate action?

|  | Daily |
| --- | --- |
|  | Several times per week |
|  | Once a week |
|  | Several times per month |
|  | Once a month |
|  | Once every few months |
|  | Once or twice a year |
|  | Never |

In which contexts have you had conversations about climate change or climate action?

*Please select all that apply.*

|  | At home |
| --- | --- |
|  | In school |
|  | At work |
|  | At a religious organization |
|  | At demonstrations (marches, vigils, rallies, etc.) |
|  | Other: |

Through what means of communication do you usually have conversations about climate change or climate action?

*Please select all that apply*

*.*

|  | Email |
| --- | --- |
|  | In-person conversation |
|  | Phone |
|  | Social media |
|  | Text chat |
|  | Video chat |
|  | Other: |

When you have conversations about climate change or climate action, how many people are usually involved (counting yourself)?

*Please select all that apply.*

|  | 2 people (one-on-one conversation) |
| --- | --- |
|  | 3-5 people (small group conversation) |
|  | 6-9 people (medium group conversation) |
|  | 10 or more people (large group conversation) |
|  | Other (please explain): |

Who do you usually talk to about climate change and climate action?

*Please select all that apply.*

|  | Acquaintances |
| --- | --- |
|  | Co-workers |
|  | Family members |
|  | Fellow activists |
|  | Friends |
|  | Neighbors |
|  | Political representatives |
|  | Strangers |
|  | Other: |

How concerned are these people about climate change?

*Please select all that apply.*

|  | Very concerned |
| --- | --- |
|  | Somewhat concerned |
|  | Slightly concerned |
|  | Not concerned |
|  | I’m not sure |

How would you describe their political orientation?

*Please select all that apply.*

|  | Very progressive |
| --- | --- |
|  | Somewhat progressive |
|  | Neither progressive nor conservative |
|  | Somewhat conservative |
|  | Very conservative |
|  | Other: |
|  | I’m not sure |

Who do you think it is most important to talk to about climate action?

*Please select up to three.*

|  | People who are very concerned about climate change |
| --- | --- |
|  | People who are somewhat concerned about climate change |
|  | People who are not sure what to think about climate change |
|  | People who are not engaging with the topic of climate change |
|  | People who are doubtful that climate change is happening |
|  | People who are dismissive of climate change (for instance, because they think it is a hoax) |
|  | Other: |

When you talk to people about climate change or climate action, how important to you are each of the following goals?

|  | Not at all important | Slightly important | Somewhat important | Fairly important | Very important |
| --- | --- | --- | --- | --- | --- |
| For them to understand that the climate is changing |  |  |  |  |  |
| For them to understand that climate change is caused by human activity |  |  |  |  |  |
| For them to understand that climate change is a serious problem |  |  |  |  |  |
| For them to understand the unjust causes and effects of climate change (for instance, that people of color and people in the global South are disproportionately impacted) |  |  |  |  |  |
| For them to understand that humans are capable of stopping climate change |  |  |  |  |  |
| For them to make lifestyle changes (dietary changes, reducing air travel, etc.) |  |  |  |  |  |
| For them to participate in collective action (strikes, lobbying, demonstrations, etc.) |  |  |  |  |  |
| For them to discuss their emotional experiences of climate change |  |  |  |  |  |
| To discuss your own emotional experiences of climate change |  |  |  |  |  |

How effective do you think your past conversations about climate change have been in achieving the goals you selected above?

|  | Very effective |
| --- | --- |
|  | Fairly effective |
|  | Somewhat effective |
|  | Slightly effective |
|  | Not effective |
|  | Counterproductive |
|  | I'm not sure |
|  |  |

In general, to what extent have your past conversations about climate change affected...

|  | Not affected | Slightly affected | Somewhat affected | Fairly affected | Greatly affected |
| --- | --- | --- | --- | --- | --- |
| ....the mindsets of the people you've spoken with? |  |  |  |  |  |
| ...the actions of the people you've spoken with? |  |  |  |  |  |

Please describe any changes in the mindsets or actions of the people you’ve spoken with.

Does having conversations about climate change generally make it easier or harder for you to continue taking action on climate change yourself?

|  | It generally makes it easier |
| --- | --- |
|  | It generally makes it harder |
|  | It doesn't make it easier or harder |
|  | It depends on the situation |
|  | I'm not sure |

If having conversations about climate change makes it easier or harder for you to continue taking action on climate change yourself, please explain why this is the case.

What barriers, if any, have prevented you from having conversations about climate change and climate action?

What barriers, if any, have prevented the people you've talked to about climate change from taking action?

In which situations do you feel the most confident talking about climate change and climate action?

In which situations do you feel the least confident talking about climate change and climate action?

**Tips for having climate conversations**

Imagine that a friend wanted to talk to people about climate change and climate action, and they asked you for tips on how to do so. What advice would you give them?

*Please be as specific as possible.*

Which of the following emotions do you think are most important to evoke when talking about climate change?

*Please select up to three.*

|  | Anger |
| --- | --- |
|  | Confidence |
|  | Determination |
|  | Hope |
|  | Joy |
|  | Empathy |
|  | Fear |
|  | Sadness/grief |
|  | Safety (i.e., making the person feel comfortable) |
|  | Guilt |
|  | Other: |

Do you have any thoughts on how to effectively communicate about climate change and climate action in a specific context or contexts? (e.g., talking with an elected official, creating a social media campaign, etc.)

*If so, please elaborate.*

Developing further research questions about climate conversations

In general, what do you consider to be the most pressing questions to be addressed by research on climate conversations?

Which research questions about climate conversations would be most helpful to you, specifically, in your organizing?

What kinds of conversations would it be most helpful for us to look at in the next phase of our study?

Possible factors to consider: the context of the conversation, the means of communication, how many people are present, and what their relationship is to each other.

What conversational goals should we focus on?

*Please select up to 3.*

|  | Educating people about the scientific consensus on climate change |
| --- | --- |
|  | Educating people about the severity of climate change |
|  | Educating people about the unjust causes and effects of climate change (for instance, that people of color and people in the global South are disproportionately impacted) |
|  | Educating people about potential solutions to climate change |
|  | Encouraging people to take part in collective action to stop climate change |
|  | Encouraging people to make lifestyle changes (for instance, to their travel, purchasing, and diet habits) to stop climate change |
|  | Other: |

How useful would each of the following resources be to you?

|  | Not at all useful | Slightly useful | Somewhat useful | Fairly useful | Very useful |
| --- | --- | --- | --- | --- | --- |
| A list of effective strategies for having conversations about climate change |  |  |  |  |  |
| A summary of findings with infographics |  |  |  |  |  |
| A video tutorial on effective strategies of having conversations about climate change |  |  |  |  |  |
| Sample scripts for conversations about climate change |  |  |  |  |  |

If you have another idea for a research product that would be useful to you, please describe it here.

**Demographic information**

In this section, we will ask questions about your demographic information. These questions will help us understand how well our survey sample has represented climate justice organizers in the U.S. and identify topics that may be of particular importance to certain demographic groups.

Which of the following categories best describes your race or ethnicity?

|  | American Indian or Alaska Native |
| --- | --- |
|  | Asian |
|  | Black or African American |
|  | Latine |
|  | Mixed race |
|  | Native Hawaiian or Other Pacific Islander |
|  | White |
|  | Other: |
|  | Prefer not to answer |

Which of the following categories best describes your gender?

|  | Genderqueer/Non-binary |
| --- | --- |
|  | Man |
|  | Woman |
|  | Other: |
|  | Prefer not to answer |

How old are you?

|  | 0-17 |
| --- | --- |
|  | 18-24 |
|  | 25-34 |
|  | 35-44 |
|  | 45-54 |
|  | 55-64 |
|  | 65-74 |
|  | 75-84 |
|  | 85-94 |
|  | 95+ |
|  | Prefer not to answer |
|  |  |

In what state do you live?

Which of the following best describes your political orientation?

|  | Very progressive |
| --- | --- |
|  | Somewhat progressive |
|  | Neither progressive nor conservative |
|  | Somewhat conservative |
|  | Very conservative |
|  | Other: |
|  | Prefer not to answer |

Which of the following best describes the political orientation of most people in your area?

|  | Very progressive |
| --- | --- |
|  | Somewhat progressive |
|  | Neither progressive nor conservative |
|  | Somewhat conservative |
|  | Very conservative |
|  | Other: |
|  | Prefer not to answer |

What was your income in the past year?

|  | $0 |
| --- | --- |
|  | $1-$9,999 |
|  | $10,000-$24,999 |
|  | $25,000-$49,999 |
|  | $50,000-$74,999 |
|  | $75,000-$99,999 |
|  | $100,000-$149,999 |
|  | $150,000 or more |
|  | Prefer not to answer |
|  |  |

Which of the following statements would you agree with?

*Please select all that apply.*

|  | My community is disproportionately impacted by climate change. |
| --- | --- |
|  | My community is not well represented in many environmental movements. |
|  | I have personally been impacted by climate change. |
|  | If a climate disaster occurred where I live, I would probably be in more danger than others. |
|  | If a climate disaster occurred where I live, I would probably face financial difficulty. |

Appendix D: Interview questions

**Background – Climate activism**

1. Please tell me about your involvement with climate justice organizing. *If you have already filled out the survey, please briefly summarize what organizations you’re part of and what your role is.*

- - Organizations: What organization(s) are you involved with, if any?
  - Role: What is your role in this organization/these organizations?
  - How long: For how long have you been involved in climate justice organizing?
  - Involvement: How did you first get involved?
  - Social justice intersections: What kinds of social justice issues do you work on in connection to climate change?
  - Media inspirations: Were there any particular conversations, books, articles, documentaries, etc. that inspired you to take action?
  - Priorities: What are your main priorities in this work?
  - Overall experience: Overall, what has your experience been like? (Rewarding? Frustrating? Draining? Etc.)
  - Motivations: What keeps you motivated to continue taking action?

2. Needs for action: What do you see as the most immediate needs for action on climate change?

- At a societal level?
- At an individual level?

3. Ways of engaging people: What do you think are the most effective ways of engaging people to take these actions?

**Climate communication experiences**

4. Kinds of communication: Let’s define “climate communication” broadly, to include face-to-face communication, e-mail, text, phonebanking, social media, opinion pieces, etc. What kinds of climate change communication do you participate in?

- Which audiences are you aiming to reach?

5. Typical climate conversations

- Participants: Who do you usually talk to about climate change? (Friends, family, co-workers, etc.)
- Settings: In what settings?
- Frequency: How often?
- Process: How do these conversations typically go?
- Goals: What goals do you have for these conversations?

6. Result: What usually happens as a result of these conversations?

- Emotional impact: How does having these conversations impact you emotionally?
- Impact on others’ actions: What actions have people taken as a result?
- Impact on own organizing: What effect do they have on your own engagement in climate action?

**Climate communication tips**

7. Effective strategies: In your experience, what strategies are effective for talking to people about climate change?

- Most effective?
- Least effective?
- Strategies to be used with specific audiences?
- Strategies for talking about climate action?
- Strategies for talking about climate justice?

8. Origin of strategies: Where did you learn about these strategies, or did you discover them yourself?

9. Target audiences: Which kinds of people do you think are most important to talk to about climate change, climate action, and climate justice?

- Why?

**Designing the next phase of the study**

*In the next phase of this study, we’re planning to analyze conversations about climate action and climate justice and identify communicative strategies that are effective within specific social contexts or across contexts.*

10. Ideas and questions: What are some ideas or questions that we should consider as we design this phase of the study?

11. Research products: What kinds of research products would be most useful to you and to other climate justice organizers? We’re considering making a list of best practices for climate change conversations and some sample scripts, but we’re open to other ideas.

12. Related research and action: Do you know of related research or activism that we should be aware of?

13. Contacts: Do you know of other climate justice organizers who might be interested in being interviewed or participating in phase II?

14. Interest in phase II (*skip if already surveyed*): Would you be interested in recording a conversation about climate action and/or climate justice for the second phase of our study?

15. Working group: Would you be interested in joining a working group to help design the second phase of the study?

Appendix E: Code list

**accessible actions**

**acknowledging others' perception of you**

Verbally recognizing an impression that you suspect someone has of you, for instance by saying “You probably think I’m a liberal, but…”

**acquaintances**

People who aren’t as close as friends, but aren’t strangers either; one possible audience to talk to about climate change

**action without hope**

The phenomenon of climate organizers continuing to be active in the movement even though they aren’t hopeful that climate change can be stopped or even meaningfully mitigated

**adaptation**

Efforts to prepare for the impacts of climate change in ways that lessen its harms

**addressing community needs**

Meeting a community’s self-identified needs, for instance through organizing or advocacy work

**affordability**

Emphasizing the low cost of climate actions, such as renewable energy, or pointing out how expensive it is to deal with uncontrolled climate change

**agency**

A person’s sense of their own power to do something in the face of climate change, or their feeling of powerlessness

**agreeing**

Taking the same stance as someone else in a conversation or explicitly saying “I agree”; often used as a strategy for building rapport

**all or nothing**

Conceptualizing climate change in terms of strict binaries, e.g., “We either completely stop climate change and live in a utopia or we face total extinction”; black-and-white thinking

**amplifying**

Uplifting the voices of others, particularly oppressed voices

**anger**

Rage about the climate crisis, often directed at corporations, governments, wealthy people, etc.; sometimes called eco-anger

**anxiety**

Worry about the climate crisis; sometimes called eco-anxiety

**apathy**

Not caring about climate injustice; often comes up in the context of asking how to talk to people who don’t care

**art/music**

Incorporating art or music into climate action, often as a way to get attention and attract people to actions

**articles**

Scientific articles and opinion pieces about climate issues

**authenticity**

Being sincere or genuine when having conversations about climate issues

**bad news**

Talking about climate change often involves delivering bad news to people; how should we deliver this bad news?

**bandwagon effect**

The tendency for people to start doing something just because others are; can be harnessed to build climate movements. Also known as “social proof.”

**barriers to action**

Factors that prevent people from taking action on climate change (and how to overcome them)

**be upfront**

The strategy of clearly stating your intentions, identity, etc. at the beginning of the conversation rather than hiding these things or leaving them unsaid

**Black**

Belonging to a racial group with brown or black skin, especially of African origin; often frontline communities

**burnout**

Exhaustion from overextending yourself, especially in climate action contexts

**canvassing**

Contacting people (usually in a specific region) to sway their opinions, often in order to get them to vote or take another political action

**capacity**

An individual’s or organization’s ability to carry out certain types of work

**capitalism**

A market-based economic system based on private ownership of the means of production; often discussed as one of the main causes of climate change

**care work**

Labor that supports and nurtures others, such as childcare, providing transportation, cooking, providing emotional support, etc.

**classmates**

Fellow students; one possible audience for climate conversations

**clear expectations**

The strategy of specifically telling people what kinds of climate actions they will be asked to do, how much time they will have to commit, etc.

**co-benefits**

Good things that happen as a result of climate action other than the main benefit of stopping or slowing climate change, such as green jobs and better health

**coalition**

Organizing cooperatively across organizations

**collective action**

Taking action on climate change as part of a coordinated community or social movement rather than doing it alone

**colonialism**

The control and oppression of Indigenous peoples by foreign settlers; often identified as a driver of climate change

**community gardens**

Spaces where community members can grow plants together; often used as a way to build community and attract people to climate movements

**community self-determination**

An impacted community making its own decisions about climate issues rather than those decisions being imposed by outsiders

**compassion**

Empathy (either for the other person in the conversation or for people impacted by climate change)

**concerned but inactive**

The segment of people who are worried about climate change but not currently taking collective action

**confidence**

Feeling that you can communicate well about climate issues

**conflict**

Disagreements about climate issues (can be between people with very different ideological views or similar ones)

**connection to broader movement**

A sense of being part of something bigger than your own climate action context

**connection to the land**

Feeling/being in close relationship with the local natural world

**conservation**

Environmentalism focused on protecting non-human species and ecosystems

**conservative**

Right-wing/Republican audiences

**COP**

The United Nations Climate Change Conference of the Parties (UNFCCC)

**core group**

The idea that you only need a small group of highly motivated and dedicated people to bring about change

**corporate campaigning**

Campaigns directed at getting corporations to change their policies and practices; can target an individual (such as a CEO) or the corporation as a whole

**corporations**

Large businesses, often identified as one of the drivers of climate change

**correcting misinformation**

Exposing falsehoods about climate issues, for instance by inoculating people against them

**COVID impacts**

How COVID has impacted climate organizing and the general public’s attitudes towards climate change

**coworkers**

People you work with (can be fellow activists if you are a professional organizer); one possible audience for climate conversations

**deep canvassing**

Canvassing that uses in-depth conversations and relationship-building in order to build movements over time rather than just mobilizing people for one action

**demonstrations**

Climate marches, rallies, protests, vigils, etc.

**deniers**

People who deny the reality of human-caused climate change or the need for appropriate action

**despair**

Lack of hope about the climate crisis

**direct action**

Action that aims to have an immediate impact, such as boycotts, strikes, blockades, sit-ins; often involves civil disobedience

**disappointment**

Feeling crestfallen when climate action fails to have the desired effects

**disasters**

Climate disasters and extreme weather such as fire, floods, tornadoes, superstorms, and extreme heat

**disengaged**

People who are not very aware of climate issues or are “checked out” from thinking about climate justice; can be a result of ignorance or apathy

**diversity**

Any discourse of diversity (ethnoracial or otherwise) in a community or organization

**divestment**

A climate action strategy involving moving money away from fossil fuel investments, and encouraging others to do so

**echo chambers**

Social spaces, such as social media groups, in which people only talk to others who have similar views; can lead to polarization

**economic impacts**

The effects of climate disasters, policies, and actions on people’s financial wellbeing

**education**

Informing people about climate science, climate justice, and climate action

**efficacy**

Estimating or measuring the effects of various kinds of climate action, with the goal of becoming more effective

**EJ/CJ**

Environmental justice or climate justice (these are grouped together because many issues touch on both); the unequal causes and impacts of environmental and climate problems

**EJ/CJ denial**

Denial of the unequal causes and impacts of environmental and climate problems; for instance, thinking that “nature doesn’t discriminate” or “all humans are destroying the earth”

**elitism**

(The perception of) climate scientists and environmentalists being privileged and excluding others

**emotionality/dispassionateness**

Whether to draw heavily on emotion-rich language or to use a more dry style when communicating about climate issues

**empowerment**

Enabling others to having more power/agency to take climate action

**energy**

Energy sources, such as fossil fuels (oil, coal, and gas) versus renewable energy (wind turbines, solar power, heat pumps)

**environmentalism**

Social movements based on conserving a non-human “environment”; not the same as more recent climate justice movements, which are more focused on human rights

**excitement**

Joyful energy and anticipation related to climate action

**faith**

Climate organizing based in religious traditions and communities

**family**

Biological or adoptive family, including parents, siblings, aunts, uncles, etc.; one possible audience for climate conversations

**fear**

Terror about impending climate disasters

**feel like you have to know everything**

Would-be activists setting a high bar for themselves to be the expert on climate issues before feeling comfortable reaching out to others; can be a barrier to climate action

**foundational conversations**

Having several conversations about climate issues over time, without an immediate call to action at first; can build trust so that the person is more likely to take action later

**friends**

People in your social circle with whom you’re fairly to very close; one possible audience for climate conversations

**frontline**

Communities that are immediately impacted by climate disasters; typically low- income and majority Black, Brown, or Indigenous communities

**frustration**

Exasperation with failures and setbacks related to climate action

**fun**

Joyful elements of climate action; a good way to attract people to events

**fundraising**

Asking for money to support climate action; a possible conversational context

**future generations**

Talking about the impacts of climate change on today’s youth or people not yet born

**future impacts**

Talking about the effects that the climate crisis will have which haven’t happened yet

**global action**

Taking action on climate change at a global level, through participation in global movements and/or policymaking or global aid

**global impacts**

The effects of climate change as seen from a global perspective, particularly including its unequal effects on the global north and the global south

**go to someone's home**

Literally meeting someone at their house; strategy for setting the scene for a climate conversation

**grassroots organizing**

Building political and social power among impacted communities

**green infrastructure**

Any environmentally friendly infrastructure, such as renewable energy facilities, transport that runs on renewable energy, parks, forests, rooftop gardens, sustainable drainage systems, rivers, canals, etc.

**green jobs**

Jobs in environmentally friendly industries, including renewable energy infrastructure

**Green New Deal**

A proposed overarching vision for policies that would provide people with jobs related to addressing climate change

**greenwashing**

Making products or practices that are not environmentally friendly appear to be so

**guiding newbies**

Helping new climate activists figure out how they fit into existing climate organizing efforts

**guilt**

A feeling of personal responsibility, remorse, and self-reproach for climate harms

**health impacts**

The effects of climate change, and action to stop it, on human health (including physical and mental health)

**heroism**

Seeing yourself as a hero for participating in climate action

**highlighting successes**

Emphasizing past successes in climate organizing, whether from your own organization or another one

**hope**

Belief that a good climate outcome is possible

**humanization**

Creating a warm, personal tone rather than a cold, robotic one

**humility**

Recognizing your own limitations with regards to climate action and decision-making

**humor**

Joking about the climate crisis, or talking about it in a light, playful tone

**identity**

Your sense of self in relation to the climate crisis, e.g. seeing yourself as an activist or a consumer

**immigrant**

People who have immigrated to the US; often members of frontline communities

**importance of language**

Comments about how and why language (including word choice, choice of language or dialect, speech style, and conversational style) matters in the context of climate organizing

**in-person conversations**

Conversations with some or all participants physically present together (as opposed to conversations over the phone, Zoom, email, or social media)

**inclusivity**

(An organization) being welcoming to a wide range of people

**Indigenous**

People from ethnoracial groups that were present in the area pre-colonialism; often at the frontlines of the climate crisis

**indirect mentions**

Raising the topic of climate change in an indirect way, such as by talking about the weather; “side doors” into climate conversations

**inertia**

The tendency for people and organizations to keep doing what they’ve been doing (even when they have reason to change)

**informing**

Telling people facts about the climate crisis, climate justice, and climate action

**intelligibility**

Expressing things in a way that is easily understandable to the target audience

**intercultural**

Conversation across cultural groups

**intersectionality**

How climate (in)justice is intertwined with other forms of social (in)justice, such as racism and income inequality

**invitation/call to action**

“Making an ask”; asking someone to participate in a specific form of climate action

**just transition**

Moving from fossil fuels to renewable energy in a way that benefits impacted workers rather than harming them

**labor**

Workforce organizing, including unions

**lack of discussion**

People not talking about climate change, which often leads to them underestimating the number of other people who are concerned; also known as the “spiral of silence”

**lack of free time**

Not having enough time to engage in climate action due to other responsibilities (work, family, etc); a barrier to climate action

**ladder of engagement**

An organizing system in which you ask people to do a small, easy action first, then gradually ask them to do harder and more intense actions until they ultimately take on a central role in the organization

**language variety**

A language or dialect (in which you could have climate conversations)

**Latine**

Latina, Latino, or Latinx; often frontline communities

**learning from other contexts**

Adapting insights from climate organizing in other geographic areas and/or times in order to improve your own organizing

**legal action**

Taking climate action through legal means, such as suing fossil fuel companies

**letting people reach their own conclusions**

Giving people information about the climate crisis, then allowing them to work through their own thoughts and feelings about climate issues rather than pushing your view on them

**lifestyle changes**

Behavioral changes that an individual can make within their own life, such as recycling, composting, and buying less or different products; typically centered on changes to consumption. Some of these same behaviors could be part of collective action through social movements focused on them (like Zero Waste), but they are not focused on policy change, and implicitly place the blame for the climate crisis on everyday people.

**listening**

Paying close attention to what your conversational partner is saying

**lobbying**

Contacting a decision maker, such as a political representative or employer, in order to sway them to make decisions that benefit climate issues

**local action**

Climate undertaken at the local level, i.e. in the nearby town, city, county, or state

**local impacts**

Effects of the climate crisis at the local (town, city, county, state) level

**love**

Social feeling of intense warmth and closeness related to climate action

**meaningful action**

Climate actions that feel meaningful to the people undertaking them, for instance because they feel effective, in-depth, or centered on justice

**media representation**

How media sources, such as newspapers, TV, etc., represent climate issues and climate activists

**meeting people where they're at**

Accommodating to focus on another person’s values, interests, or views

**meetings**

Climate activists convening, usually either in-person or over Zoom

**morality**

Framing climate issues in terms of moral arguments (i.e. what is the right thing to do)

**motivation**

A person’s reasons for engaging in climate action

**moveable middle**

People who aren’t yet active in climate movements, but who also aren’t dead set against taking action

**narrow focus**

Only addressing climate issues, or only addressing one aspect of climate issues, rather than focusing on a wide range of related issues

**national action**

Climate action at the national (U.S.-wide) level

**neighbors**

People who live next door to you; one possible audience for climate conversations

**normalcy pressure**

The pressure to behave in a way that feels normal and appears normal to others, or to believe or feel that the world is “normal” despite indications that things are going badly wrong; can be a deterrent to climate action

**normalization**

Making something that seems weird to society not seem weird anymore; for instance, “normalizing climate conversations” entails making it seem ordinary to have a conversation about climate change

**offering food**

Giving food to people at events or during climate conversations

**old people**

Depending on context, this can mean elders (60+) or adults; anyone contrasted with youth

**one-on-one conversations**

Conversations involving only two people; sometimes referred to as “one-on-ones”

**open-mindedness**

Being willing to consider other positions than your own when talking about climate issues

**opportunity for change**

Conceptualizing climate change as an opening for beneficial social change

**other priorities than climate**

Having other things to worry about than climate issues, such as economic security, police violence, etc.

**overwhelm**

Being overcome by the scope of the climate crisis, by the amount of possible climate actions, or by other concerns and responsibilities

**peer-to-peer**

Conversations with others who are similar to you in life stage or profession (for instance, a doctor talking to other doctors, or students talking to other students)

**personal experience**

Having personally lived through an event related to climate issues

**personalization/tailored messenging**

Adapting your talking points to a specific individual or group in order to resonate with them

**Phase 2**

Participants’ ideas about which conversational contexts to research for the second phase of the project

**phone banking**

A type of climate conversation in which you call up people (usually strangers) to convince them to take a political action or change their stance on something

**polarization**

The drastic separation between opposing views, and between groups of people who hold those views; often used to talk about the separation of Republicans and Democrats

**political action**

Climate action focused on effecting political change

**politicization**

Making climate change into a politically divisive issue

**pollution**

Making air, water, or land dirty and unsafe

**poverty/wealth**

Any reference to poor people, rich people, or income inequality

**prefigurative**

Acting or living the way you want everyone to act or live in the future

**privilege**

Unearned benefits accrued through an unjust system, such as the benefits conferred by Whiteness or generational wealth

**questions**

Asking questions in the course of climate conversations

**racial (in)justice**

Any mention of the social justice issues relating to race

**radical**

Being in favor of swift and drastic, rather than incremental, sociopolitical climate action

**read between the lines**

Interpreting the deeper meaning or emotions behind what someone appears to be saying

**refugees**

Climate refugees; immigrants fleeing climate disaster

**region**

Surrounding geographical area as a factor in how to talk about climate change

**relatability**

How easy it is for people to personally identify with a story or message

**relationship-building**

Getting to know a conversational partner in a more than superficial way

**relationships with other activists**

Friendships, partnerships, or a sense of community belonging with other climate activists

**repetition**

Saying the same message several times to the same person, either in a single interaction or spaced out over time

**rephrasing**

Listening to what someone said and repeating it back to them in a different way to show or check that you understood them

**research products**

Proposed materials resulting from this research project, such as scripts, informational videos, pamphlets, etc.

**resilience**

People’s ability to persevere through climate hardships

**respectability politics**

Having to behave or speak in a certain way that is deemed professional or socially acceptable in order to be taken seriously; particularly in the case of Black Americans having to adhere to dominant White norms

**rigidity**

Sticking to a fixed conversational template or script instead of having a free-flowing conversation

**role modeling**

Demonstrating desired climate actions for others

**rootedness**

Being deeply connected to one’s local community, often as a result of having lived there for a long time and having strong social ties

**rural/urban**

Any mention of whether an area is rural, urban, or suburban

**sadness/grief**

Being sad or depressed about climate issues; “eco-grief”

**savior complex**

Seeing yourself as a hero who will save disadvantaged people (without their input); often an attitude held by White people

**science communication**

Conveying climate science to various audiences

**scripts**

Written examples of climate conversations that can be used as guides for future conversations

**self-selecting group**

Only people who are interested in climate action showing up to participate in an organization

**self-sufficiency**

Being able to meet your basic needs without aid from others; often used in the context of energy self-sufficiency (“living off the grid”)

**sensationalism**

Talking about climate issues in showy or dramatic tones

**sense of purpose**

Having a clear idea of what to do in life and why; can be provided by climate action

**silencing**

Forcing others not to speak, denying them a platform, or drowning out their voices (in relation to climate issues)

**social media**

Any form of digital social connection, including Facebook, Twitter, Instagram, TikTok, WhatsApp, YouTube, etc.

**spatiotemporal distancing**

Making the climate crisis seem far away in time or space

**speak their language**

Adapting your speech style, word choice, or choice of language variety to an audience

**specificity**

Naming particular climate facts, climate impacts and climate actions instead of speaking in general terms

**sporadic participation**

Climate activists only taking action once in a while; can be used as a strategy to combat burnout

**state-level action**

Climate action at the state level, such as seeking to influence state-wide policies

**statistics**

Using numbers to talk about climate change, e.g. by saying “71% of emissions are caused by 100 companies”

**storytelling**

Telling a narrative about climate change; can be either a personal narrative or a bigger-picture narrative

**strangers**

People you don’t know at all; one possible audience for climate conversations

**strategy conversations**

Conversations about how to take climate action; usually held with other activists

**succinct**

Being concise when talking about climate change; making your point in only a few words or in a short amount of time

**sustaining action**

Supporting climate activists in taking action over the long term

**talking to/talking at**

The difference between talking to someone in an interactive way (including listening to them) and monologuing at/lecturing to someone

**tangibility**

Concreteness of climate actions or climate impacts; being able to see, hear, touch, or otherwise immediately perceive them

**trainings/workshops**

Any event aimed at teaching climate activists to improve their skills

**trust**

Belief that another person is honest and/or has your best interests at heart; cultivated as part of successful climate conversations

**turnout**

How many people show up to climate actions

**unsure what to do**

People not knowing what climate actions to take; can be a barrier to action

**urgency**

The sense that action must be taken quickly to stop the climate crisis

**values**

What matters to someone, often in a moral sense; a good thing to be aware of in order to have climate conversations

**video**

Any kind of video media related to climate issues, including documentaries, short films, YouTube videos, TikToks, and Instagram or Snapchat films; useful jumping off points for climate conversations

**visuals**

Images, charts, or diagrams that help people understand climate messages

**vulnerability**

Presenting oneself in a way that exposes sensitive parts of you and could allow others to hurt you; used as a strategy to build trust in climate conversations

**what not to say**

Words, phrases, or framings to avoid when talking about climate issues

**Whiteness**

The ethnoracial category of being White, and the systemic privilege conveyed by it

**who to talk to**

Who to involve in climate conversations; who to reach out to with messages about climate issues

**word of mouth**

People sharing a message beyond an initial interaction, and thus spreading it to a wider audience

**work parties**

Events where people come together to physically work on something related to the environment, such as beach clean-ups or tree planting sessions

**youth**

Young people, ranging from children to young adults; anyone contrasted with old people

**Zoom**

A video chat service often used in climate organizing

1. United States Census Bureau. Race and ethnicity in the United States: 2010 census and 2020 census. <https://www.census.gov/library/visualizations/interactive/race-and-ethnicity-in-the-united-state-2010-and-2020-census.html> [↑](#footnote-ref-1)
2. The percentages in this table do not sum to 100 because several participants held multiple roles in their organization(s). [↑](#footnote-ref-2)
